# Morphological, Physiological, and Structural Responses of Two Species of *Artemisia* to NaCl Stress

**DOI:** 10.1155/2013/309808

**Published:** 2013-10-22

**Authors:** Zhi-Yong Guan, Yi-Ji Su, Nian-Jun Teng, Su-Mei Chen, Hai-Nan Sun, Chu-Ling Li, Fa-Di Chen

**Affiliations:** ^1^College of Horticulture, Nanjing Agricultural University, Nanjing 210095, China; ^2^Jiangsu Province Engineering Lab for Modern Facility Agriculture Technology & Equipment, Nanjing 210095, China

## Abstract

Effects of salt stress on *Artemisia scoparia* and *A. vulgaris* “Variegate” were examined. *A. scoparia* leaves became withered under NaCl treatment, whereas *A. vulgaris* “Variegate” leaves were not remarkably affected. Chlorophyll content decreased in both species, with a higher reduction in *A. scoparia*. Contents of proline, MDA, soluble carbohydrate, and Na^+^ increased in both species under salt stress, but *A. vulgaris* “Variegate” had higher level of proline and soluble carbohydrate and lower level of MDA and Na^+^. The ratios of K^+^/Na^+^, Ca^2+^/Na^+^, and Mg^2+^/Na^+^ in *A. vulgaris* “Variegate” under NaCl stress were higher. Moreover, *A. vulgaris* “Variegate” had higher transport selectivity of K^+^/Na^+^ from root to stem, stem to middle mature leaves, and upper newly developed leaves than *A. scoparia* under NaCl stress. *A. vulgaris* “Variegate” chloroplast maintained its morphological integrity under NaCl stress, whereas *A. scoparia* chloroplast lost integrity. The results indicated that *A. scoparia* is more sensitive to salt stress than *A. vulgaris* “Variegate.” Salt tolerance is mainly related to the ability of regulating osmotic pressure through the accumulation of soluble carbohydrates and proline, and the gradient distribution of K^+^ between roots and leaves was also contributed to osmotic pressure adjustment and improvement of plant salt tolerance.

## 1. Introduction

Salinity is one of the major environmental stresses affecting crop productivity. Excessive irrigation and poor drainage facilities are the main factors causing soil salinity in agricultural lands, and about one-third of world irrigated land is being affected by soil salinity [1, 2]. Injury resulting from salinity is mainly symbolized as ion toxicity, osmotic stress, and nutritional imbalance [3]. NaCl stress leads to higher concentration of Na^+^ in plant organs, and the excessive accumulation of Na^+^ can inhibit plant growth and development [4]. To maintain normal physiological metabolism, the plant restricts Na^+^ entrance through selective absorption by roots, which promotes the efflux and compartmentation of Na^+^, and maintains high ratio of K^+^/Na^+^ balance [5]. Thus, the mechanism of salt tolerance for most of crops is to keep a low concentration of Na^+^ and high absorption of K^+^ [6]. Previous research on ion distribution in plants under salt stress has been conducted on soybeans (*Glycine max*), wheat (*Triticum aestivum*), cotton (*Gossypium hirsutum*), sorghum (*Sorghum bicolor*), *Solanum* sp., and *Cucumis sativus* [7–9], while little information is available on *Chrysanthemum *and its related genera.

*Artemisia*, belonging to Compositae, is closely related to *Chrysanthemum*. The species in *Artemisia* have plenty of valuable characters that *Chrysanthemum* cultivars do not have, such as cold tolerance and aphid resistance [10, 11]. Therefore, many *Artemisia* species are very important germplasm resource during *Chrysanthemum* breeding with the aim of improving its biotic and abiotic resistance. The collection, evaluation, and selection of wild species of *Artemisia* are of great significance for future breeding of *Chrysanthemum.* However, few studies have been conducted to assess salt tolerance in this genus. Therefore, it is very necessary to evaluate their salt tolerance and investigate the mechanism involved in salt tolerance.

*Artemisia scoparia* and *A. vulgaris *“Variegate” are two main species in *Artemisia* and widely distributed in China [10, 11]. We therefore used the two species as experimental materials in this study to investigate their morphological, physiological, and structural responses to NaCl stress. The aim of this study is to evaluate their salt tolerance and related mechanism of salt tolerance and obtain salt-tolerant species for salt-tolerant breeding of *Chrysanthemum* in the future. 

## 2. Materials and Methods

### 2.1. Plant Material

*Artemisia scoparia* and *A. vulgaris* “Variegate” were obtained from the Chrysanthemum Germplasm Resource Preserving Centre, Nanjing Agricultural University, China (32°05′ N, 118°90′ E). 

### 2.2. NaCl Treatment

Shoot cuttings of *A. scoparia* and *A. vulgaris* “Variegate” were rooted and grown in a sand bed from the beginning of April 2012. Rooted seedlings at 6-7 leaf stage were selected and then transplanted into 300 mL plastic pots filled with quartz sand that has been washed by acid and water successively. Hoagland nutrient solution was provided to plants in a circulation case (volume = 23.4 L), with aeration for 24 h/d. After 1 week, salt treatment was performed by supplementing the nutrient solution with 200 mmol·L^−1^ NaCl. A set of plants growing on Hoagland solution alone was kept as a control (CK). Plants were treated under hydroponic cultivation for 14 days; the stress treatment solutions were renewed every 3 days. Each treatment had 15 plants. All the plants were maintained in a greenhouse at 160 mol·m^−2^·s^−1^ PAR, 12 h photoperiod, average temperature of 25°C and relative humidity of 70%.

### 2.3. Determination of Physiological Parameters

 Chlorophyll contents were determined by ethanol extraction colorimetry. 0.2 g fresh leaves were put into mortar and grinded with the mixture of leaves, quartz sand, calcium carbonate powder, and 2-3 mL 95% ethanol. After the volume was determined, the absorbance values were measured under 665 nm, and 649 nm. The contents were calculated according to the following formula:

(1) $$\begin{array}{c} C_{a}=13.95A_{665}-6.88A_{649},\\ C_{b}=24.96A_{649}-7.32A_{665}. \end{array}$$

Malondialdehyde (MDA) contents were determined by Tribromoarsenazo (TBA) colorimetry. 5 mL 5% TCA was added to 0.5 g fresh leaves. The mixture was centrifuged under 3000 r/min for 10 min after grinded. 2 mL 0.67% TBA was added into 2 mL supernatant. The mixed solution was put in 100°C boiling bath for 30 min. After being cooled and centrifuged, the absorbance values were measured under 450 nm, 532 nm and 600 nm, *C* (*μ*mol/L) = 6.45(*A*_532_ − *A*_600_) − 0.56*A*_450_.

With regard to proline content, 0.5 g of fresh leaves was put into big tubes and 5 mL of 3% sulfosalicylic acid aqueous solution was added. The mixture was extracted in the boiling water for 10 min and 2 mL of them was taken into clear tubes with glass plugs. 2 mL acetic acid and 2 mL acidic-ninhydrin were added and put in the boiling bath for 30 min. After being cooled and shaken for 30 s, 4 mL toluene was added with a short period of standing. The upper liquor was centrifuged under 3000 r/min for 5 min in 10 mL tubes, and the absorbance values were measured under 520 nm. Proline contents were calculated according to the standard curve: proline content (%) = (*X* × *V*_*T*_)/(*W* × *V*_*S*_ × 10^6^) × 100.

For the content of soluble carbohydrate, the phenol method was carried out. 0.10–0.30 g of fresh leaves was taken into tubes and 5–10 mL diluted water was added. Tubes sealed with plastic films were extracted in boiling water twice, 30 min each time. After filtration and volume determined, the absorbance values were measured under 485 nm, and contents were calculated according to the standard curve: soluble carbohydrate content (%) = (*C* × *V*_*T*_ × *N*)/(*W* × *V*_*S*_ × 10^6^) × 100.

In ion measurement, the seedlings were washed and divided into four parts: roots, stems, middle leaves (the third and fourth mature leaves counting from the apex) and upper leaves (the newly unrolled leaves after treatment). Then enzymes were deactivated under 105°C for 25 min and the dry weight of samples was measured after they were dried to constant weight under 70°C. After being grinded, the samples were put into the dryer for storage. 50 mg of dry samples; taken into tubes, then 20 mL of water was added and vortexed. The samples were filtered into 25 mL volumetric flask after staying in boiling water bath for 1.5 h. The contents of K^+^, Na^+^, Ca^2+^, and Mg^2+^ in the nutrient solutions of each treatment were measured by Optimal 2100 DV Inductive Coupling Plasma Emission Spectrograph (Perkin Elmer Co.).

The selective ratios of K^+^ and Na^+^ absorption and transport (*S*_K,Na_) were calculated as follows:

- ion absorption *S*_K,Na_ = root ([K^+^]/[Na^+^])/medium ([K^+^]/[Na^+^]);
- ion transport *S*_K,Na_ = sink organ ([K^+^]/[Na^+^])/source organ ([K^+^]/[Na^+^]).

### 2.4. Transmission Electron Microscopy

 Tissue samples for ultrastructural observation were taken from the middle section of the second and third leaf counting from the apex. Leaf segments with approximately 0.5 mm in length were fixed with 2.5% glutaraldehyde in phosphate buffer (pH 7.2) for 3 h at room temperature and then postfixed with 1% OsO_4_ in the same buffer with the addition of sucrose (25 mg/mL) for 2 h. Subsequently, the samples were dehydrated in a series of gradient ethanol (30, 40, 50, 60, 70, and 96%), acetone, and propylene oxide. The samples were embedded in Epon 812 and polymerized for 3 days at temperature rising from 37 to 60°C. The sections were produced using an LKB-111 microtome (Sweden) and stained with a saturated solution of aqueous uranyl acetate for 10 min at 60°C and then for 10 min with aqueous lead citrate. Preparations were examined under a transmission electron microscope (JEOL Ltd, Tokyo, Japan) [12, 13].

### 2.5. Statistical Analysis

The data were subjected to a one-way analysis of variance and statistical significance (*P* < 0.05) of differences among means was judged by Duncan's New Multiple Range Test using the SPSS software 16.0 (SPSS Inc., Chicago, IL, USA).

## 3. Results

### 3.1. Morphological Characteristics

**Figure 1:**
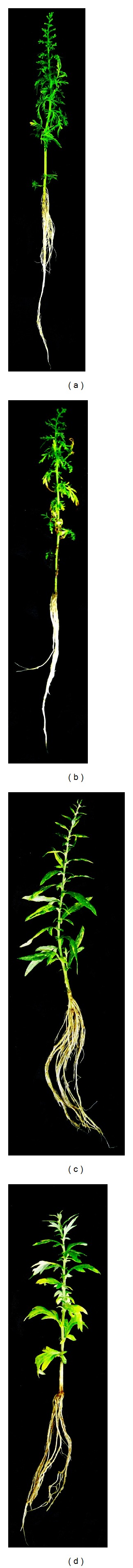
Morphological response of *A. scoparia* and* A. vulgaris* “Variegate” to salt stress. (a), (c) The plants of *A. scoparia* and* A. vulgaris* “Variegate” grown under controls, respectively. (b), (d) The plants treated with NaCl, respectively.

Compared with* A. vulgaris* “Variegate,” *A. scoparia* was more sensitive to NaCl treatment. For example, most of *A. scoparia* leaves withered and became brown after NaCl stress, whereas *A. vulgaris* “Variegate” was seldom affected by NaCl and only a few leaves turned yellow (Figure 1).

### 3.2. Chlorophyll Content

**Figure 2:**
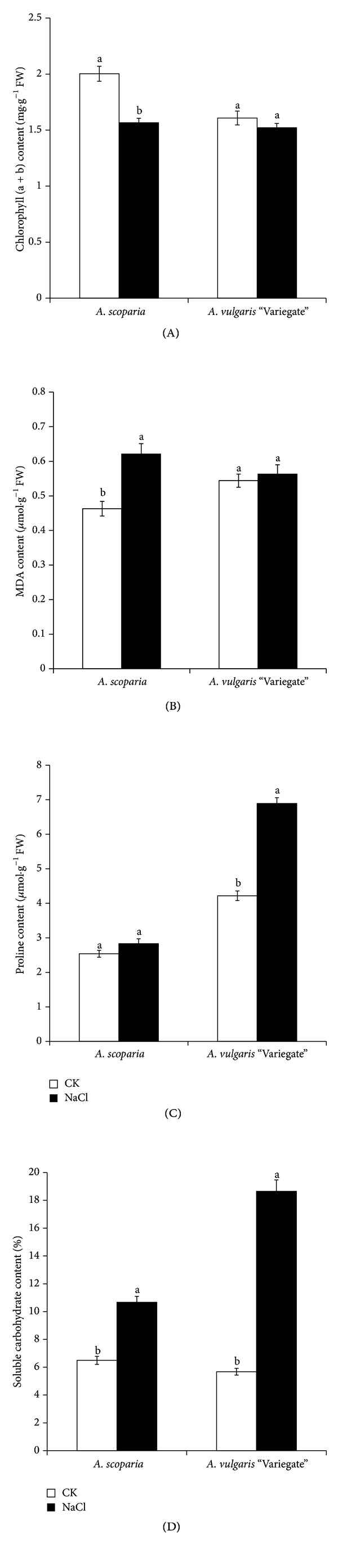
Effect of NaCl on contents of chlorophyll (a + b), MDA, proline, and soluble carbohydrate content in leaves. CK: 0 mmol·L^−1^ NaCl treatment; NaCl: 200 mmol·L^−1^ NaCl treatment. The different letters represent significant difference at *P* < 0.05 between CK and NaCl by Duncan's New Multiple Range Test. Error bars represent standard errors.

NaCl stress significantly reduced the chlorophyll content of *A. scoparia* to 78% of the untreated control plants, but the chlorophyll content of *A. vulgaris* “Variegate” only had a slight reduction of 5% relative to the control. The results indicate that *A. vulgaris* “Variegate” was less affected by NaCl stress compared with *A. scoparia* (Figure 2(A)).

### 3.3. MDA Content

 NaCl treatment significantly increased MDA content in the leaves of *A. scoparia*, but MDA content in the leaves of *A. vulgaris* “Variegate” slightly increased under NaCl stress. For instance, MDA content in the leaves of *A. scoparia* increased by around 34% after NaCl treatment, while the level of increase in *A. vulgaris* “Variegate” was only about 3%. As MDA content in the leaves is usually proportional to the damage level of plant membrane, *A. vulgaris* “Variegate” was more tolerant to salt stress than *A. scoparia* (Figure 2(B)).

### 3.4. Proline Content

Proline content increased slightly in the leaves of *A. scoparia* but significantly in the leaves of *A. vulgaris* “Variegate”. Compared with the controls, NaCl treatment resulted in a slight increase of about 11% in *A. scoparia* and a significant increase of around 63% in *A. vulgaris* “Variegate”, respectively (Figure 2(C)). Because proline is one of the most important substances involved in osmoregulation and reducing membrane injury when plants are subject to abiotic stresses including salt stress, it can help increase plant tolerance to abiotic stresses. Therefore, the results presented here indicate that *A. vulgaris* “Variegate” may have a higher tolerance to salt stress than *A. scoparia*. 

### 3.5. Soluble Carbohydrate Content

 Soluble carbohydrate content significantly increased in leaves of both* A. scoparia* and *A. vulgaris* “Variegate” under salt stress, but there is a higher increase level in the latter than in the former species. For example, NaCl treatment led to an increase of about 64% in soluble carbohydrate content in leaves of *A. scoparia* but around 228% in the leaves of* A. vulgaris* “Variegate”, relative to the controls (Figure 2(D)). 

### 3.6. Allocation of Na^+^, K^+^, Ca^2+^, and Mg^2+^

NaCl stress significantly increased Na^+^ content in different parts of both species. However, there is a higher level of increase in Na^+^ content in roots, stems, and upper leaves of *A. scoparia* than in *A. vulgaris* “Variegate”. For instance, Na^+^ content in roots, stems, and upper leaves of *A. scoparia* was 8.34, 5.24, and 9.46 times of that in the corresponding parts of this species under the control, respectively, whereas the data were 4.67, 2.09 and 3.90 (Table 1). In addition, after NaCl treatment, Na^+^ content in all the parts of *A. scoparia*, especially leaves, was much higher than those of *A. vulgaris* “Variegate”. 

NaCl stress signicantly decreased K^+^ content in roots and stems of both species but did not significantly affect K^+^ content in leaves of the two species. In addition, there were higher reductions in K^+^ content in roots and stems of *A. scoparia* than in those of *A. vulgaris* “Variegate” after both species were exposed to NaCl stress. For example, the ratios (NaCl : CK) of K^+^ content in roots and stems of *A. scoparia* were 0.46 and 0.74, respectively, and the corresponding data for those of *A. vulgaris* “Variegate” were 0.60 and 0.82, respectively. However, the ratios (NaCl : CK) for middle and upper leaves were 0.94 and 0.97 in *A*.* scoparia* and 1.09 and 1.00 in *A. vulgaris* “Variegate” (Table 2). 

In contrast to other cations, levels of Ca^2+^ in the plant organs of both species grown in absence of NaCl were similar, except that the roots of *A. vulgaris* “Variegate” had levels about three times higher than those of *A. scoparia*. When the two species were exposed to salt treatment, Ca^2+^ levels decreased in roots and stems of both species but slightly increased in middle leaves and upper leaves (Table 3). 

There were significant reductions in Mg^2+^ content in roots of both *A. scoparia* and* A. vulgaris* “Variegate” after salt stress, but Mg^2+^ content in stems and leaves of both species was not significantly affected by NaCl stress. In addition, there was a higher reductions in Mg^2+^ content in roots of *A. scoparia* (NaCl:CK is 0.28) than in those of *A. vulgaris* “Variegate” (NaCl:CK is 0.63), indicating that Mg^2+^ content in roots of *A. scoparia* was more easily affected by NaCl than that of *A. vulgaris* “Variegate” (Table 4).

### 3.7. Ratios of K^+^/Na^+^, Ca^2+^/Na^+^, and Mg^2+^/Na^+^ in Root, Stem, and Leaves

 The ratios of K^+^/Na^+^ in all organs of the two species decreased dramatically when they were subjected to NaCl stress. In addition, the ratios of K^+^/Na^+^ in leaves of *A. vulgaris* “Variegate” were significantly higher than those in leaves of *A. scoparia* under NaCl stress, but there was no significant difference in ratios of K^+^/Na^+^ for roots or stems between *A. scoparia* and *A. vulgaris* “Variegate” (Table 5). Compared with CK, the transport selectivity of K^+^/Na^+^ (designed as *S*_K,Na_) was remarkably higher in each organ of *A. vulgaris* “Variegate” treated with NaCl, whereas in *A. scoparia*, only *S*_K,Na_ from root to stem increased after NaCl treatment. In addition, *S*_K,Na_ in each organ of *A. vulgaris* “Variegate” was higher than that in the corresponding parts of *A. scoparia* after NaCl treatment (Table 6).

There was a significant decrease in the ratios of Ca^2+^/Na^+^ in all organs of both species that were exposed to NaCl stress. In addition, the ratios of Ca^2+^/Na^+^ in all organs of *A. vulgaris* “Variegate” were much higher than those in the corresponding parts of *A. scoparia *under NaCl stress (Table 7). The transport selectivity of Ca^2+^/Na^+^ (designed as *S*_Ca,Na_) from roots to stems increased significantly in *A. scoparia* under salt stress but only slightly from stem to middle leaves and actually decreased from stem to upper leaves. The transporting *S*_Ca,Na_ pattern in *A. vulgaris* “Variegate” was different. There was a 35% decrease from roots to stems and substantial increase from stems to middle leaves and from stems to upper leaves (Table 8).

The ratios of Mg^2+^/Na^+^ in all organs of both species were decreased under NaCl stress. In addition, the ratios of Mg^2+^/Na^+^ in all organs of *A. vulgaris* “Variegate” were much higher than in those of *A. scoparia* under salt stress, though there was no significant difference except for upper leaves (Table 9). Compared with CK, transporting *S*_Mg,Na_ both from roots to stems and from stems to middle leaves in *A. scoparia *was remarkably higher, whereas there was a slight decrease from stems to upper leaves. In *A. vulgaris* “Variegate”, transporting *S*_Mg,Na_ of all parts was higher than CK (Table 10). 

### 3.8. Leaf Ultrastructure

**Figure 3:**
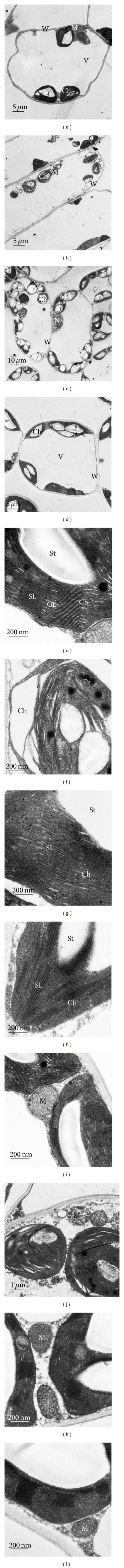
Effect of NaCl stress on leaf ultrastructure of *A. scoparia* and *A. vulgaris* “Variegate”. Mesophyll cells of untreated control (a) and NaCl-treated (b) plants of *A. scoparia* and of *A. vulgaris* “Variegate” ((c) and (d), resp.). Chloroplasts ((e)–(h)) and mitochondria ((i)–(l)) of the same plants in the same order. Abbreviations: Ch: chloroplast; M: mitochondria; W: cell wall; N: cell nucleus; V: vacuole; St: starch grain; SL: stroma lamelle; GL: grana lamelle; Pr: peroxisome.

Mesophyll cells of *A. scoparia* were severely damaged under NaCl stress (Figures 3(a) and 3(b)), whereas no apparent damage was observed in *A. vulgaris* “Variegate” (Figures 3(c) and 3(d)). However, NaCl treatment reduced the number of chloroplasts in mesophyll cells of both species. Chloroplast ultrastructure of *A. scoparia* was more severely affected by NaCl stress relative to *A. vulgaris* “Variegate”. For example, the chloroplasts of *A. scoparia* mesophyll cells were seriously injured departing from the normal appearance with clear stroma lamellae and large starch grains (Figure 3(e)) to malformed, rounded shape with large interior space and disorganized lamellae (Figure 3(f)). In contrast, chloroplasts of *A. vulgaris* “Variegate” remained relatively normal under the treatment of NaCl, except for smaller starch grains and thinner stacking grana (Figures 3(g) and 3(h)). In addition, mitochondria of *A. scoparia* were severely affected by NaCl stress, while those of *A. vulgaris* “Variegate” were seldom influenced. Mitochondria in untreated plants of both species had a normal appearance with intact membranes and cristae (Figures 3(i) and 3(k)). Mitochondria of *A. scoparia* exposed to NaCl stress became vacuolated and their membranes lost the integrity (Figure 3(j)). However, mitochondria of *A. vulgaris* “Variegate” exposed to NaCl stress were not seriously affected.

## 4. Discussion

The salt-tolerant ability of plants is controlled by multiple genes that are involved in numerous physiological processes [1, 2, 14–16]. Among them, photoassimilation is inhibited under salt stress and the degree of reduction in photoassimilation is positively proportional to stress strength. The decrease in chlorophyll content under salt stress is due to the increase in activity of chlorophyllase promoting its degradation [17, 18]. Our results are in accordance with the negative impact of salt stress in chlorophyll content and illustrate well how a species with less sensitivity to salinity is able to maintain its chlorophyll levels when exposed to a high concentration of NaCl.

The dynamic balance between production and removal of reactive oxygen species (ROS) *in vivo* can be destroyed by salt stress [19, 20]. Increase in ROS level leads to the accumulation of MDA that can result in peroxidation and delipidation of membrane lipids with a concomitant loss of membrane integrity [19, 21]. Accumulation of proline under salt stress serves as regulator for cytoplasm osmotic pressure, thus protecting the structures of membranes and enzymes [21, 22]. In this research, the differential accumulation of proline between *A. scoparia* (susceptible to NaCl stress) and *A. vulgaris* “Variegate” (salt tolerant) reflected the adaptive response to salt stress, whereas the increase in content of MDA indicated the degree of cell injury. Organic osmolytes mainly include soluble carbohydrate and organic acids. From the above conclusions, it can be illustrated that both soluble carbohydrate and proline have significant effect on regulating the osmosis.

Plant salt tolerance is determined by the absolute contents of salt ions and priority sequence of ion distribution under organic level [23, 24]. Na^+^ contents in all the organs of salt-tolerant *A. vulgaris* “Variegate” under NaCl stress were lower than in those salt-sensitive *A. scoparia*; the stems of *A. vulgaris* “Variegate” also absorbed Na^+^ with a higher degree, which alleviated Na^+^ stress to leaves. The metabolic activity of the stems is relatively lower than that of roots and leaves. Since it mainly serves as transport and support, the ion accumulation in stem can reduce the damage to functional organs [25].

K^+^ is not only a key ion relative to salt tolerance [26], but also the cation in most of higher plants and can regulate physiological functions, such as ion balance, osmosis, protein synthesis, cell turgor, and photosynthesis. Since the ionic radius and hydration energy of Na^+^ and K^+^ are similar, Na^+^ will compete the absorption and binding sites of K^+^ and lead to the inhibition of enzyme activity and metabolic process which depends on K^+^. Under salt stress, relatively high K^+^ content and K^+^/Na^+^ ratio can reduce salt damage to plants, which is essential for normal activities of organisms [27, 28]. The ability of *A. vulgaris* “Variegate” to maintain relatively high K^+^/Na^+^ ratio in all the organs under salt stress can partially explain its strong salt tolerance.

The present study illustrated that most Na^+^ was transported into aging organs, while K^+^ was transported into juvenile organs. Meanwhile, relatively high ratios of K^+^/Na^+^, Ca^2+^/Na^+^, and Mg^2+^/Na^+^ were effective to enhance salt-tolerance.

In addition, the relatively low Ca^2+^ and Mg^2+^ contents in salt-stressed plants could be attributed to the increase in Na^+^ and Cl^−^ contents [29]. Further, the decrease in Ca^2+^ and Mg^2+^ causes unstacking of thylakoid membranes which can be compensated by the supply of higher polyamines, as they have strong cationic effects and allow for the stacking of thylakoids [30, 31].

Based on the results for chlorophyll content and damage to the ultrastructure of mesophyll cells, we conclude safely that the high salinity induced stomatal closure leading to reduction in photosynthesis and biomass [32]. Among plant organelles, chloroplast is the most sensitive to salinity injury. For example, salt stress may cause membrane and thylakoid disorganization, which leads to lower photosynthetic efficiency and avoid or alleviate the photo oxidation [33]. Thus it is a mechanism implicated in an adaption to stress. Mitochondria are more stable to stress compared with chloroplast. The close distribution between mitochondria and chloroplasts may be beneficial for the usage of metabolic materials (such as CO_2_, H_2_O, and O_2_.), which will compensate for the reduction in cristae and the decrease in metabolic activity [34].

In conclusion, we systematically investigated effects of NaCl stress on morphological, physiological and structural characteristics of two species in the genus of *Artemisia* in the current suty. The results indicate that *A. vulgaris* “Variegate” is highly tolerant to salt stress, whereas *A. scoparia* is very sensitive to salt stress. Therefore, *A. vulgaris* “Variegate” can be used as the important material in *Chrysanthemum* hybridization breeding with salt-tolerant improvement in the future. In addition, some physiological and structural parameters can be used for the effective assessment of salt-tolerant ability of many wild species related to *Chrysanthemum* and even other crops. Finally, the mechanism involved in salt-tolerant ability will provide valuable information for genetic improvement of salt-tolerant cultivars by plant molecular biotechnology. 

## Figures and Tables

**Table 1:** Effects of NaCl stress on Na^+^ content in different organs of *A. scoparia* and *A. vulgaris* “Variegate.”

Cultivars	Treatment	Na^+^ content (mg·g^−1^ dry weight)			
		Roots	Stems	Middle leaves	Upper leaves
*A. scoparia *	CK	8.10 ± 0.30b	17.97 ± 0.54c	13.39 ± 0.59c	8.28 ± 0.27c
	NaCl	67.52 ± 2.18a	73.39 ± 2.21a	70.17 ± 1.58a	78.37 ± 1.94a
	NaCl : CK	8.34	4.08	5.24	9.46

*A. vulgaris *“Variegate”	CK	14.04 ± 0.52b	9.03 ± 0.51d	13.68 ± 0.36c	5.40 ± 0.15c
	NaCl	65.61 ± 2.75a	57.38 ± 2.04b	28.61 ± 1.50b	21.04 ± 0.72b
	NaCl : CK	4.67	6.35	2.09	3.90

Values (mean ± standard errors) with the different letters in the same line are significantly different at *P* < 0.05 by Duncan's New Multiple Range Test. CK: 0 mmol·L^−1^ NaCl treatment; NaCl: 200 mmol·L^−1^ NaCl treatment.

**Table 2:** Effects of NaCl stress on K^+^ content in different organs of *A. scoparia* and *A. vulgaris *“Variegate.”

Cultivars	Treatment	K^+^ content (mg·g^−1^ dry weight)			
		Roots	Stems	Middle leaves	Upper leaves
*A. scoparia *	CK	55.35 ± 1.92a	81.88 ± 1.03a	64.37 ± 2.51a	72.95 ± 1.91a
	NaCl	25.53 ± 1.32c	60.40 ± 1.54bc	60.32 ± 1.18a	70.89 ± 0.90a
	NaCl : CK	0.46	0.74	0.94	0.97

*A. vulgaris *“Variegate”	CK	37.44 ± 1.16b	63.57 ± 1.75b	58.80 ± 2.35a	75.06 ± 1.98a
	NaCl	22.44 ± 1.10c	51.92 ± 1.58c	64.23 ± 2.58a	74.83 ± 0.56a
	NaCl : CK	0.60	0.82	1.09	1.00

Values (mean ± standard errors) with the different letters in the same line are significantly different at *P* < 0.05 by Duncan's New Multiple Range Test. CK: 0 mmol·L^−1^ NaCl treatment; NaCl: 200 mmol·L^−1^ NaCl treatment.

**Table 3:** Effects of NaCl stress on Ca^2+^ content in different organs of *A. scoparia* and *A. vulgaris *“Variegate.”

Cultivars	Treatment	Ca^2+^ content (mg·g^−1^ dry weight)			
		Roots	Stems	Middle leaves	Upper leaves
*A. scoparia *	CK	18.51 ± 0.66c	32.67 ± 1.76a	32.54 ± 1.99a	22.56 ± 1.25a
	NaCl	11.73 ± 0.64d	23.56 ± 0.88bc	36.27 ± 0.97a	29.53 ± 1.15a
	NaCl : CK	0.63	0.72	1.11	1.31

*A. vulgaris *“Variegate”	CK	62.89 ± 2.01a	31.44 ± 1.96ab	31.61 ± 1.21a	22.85 ± 1.48a
	NaCl	48.03 ± 1.53b	21.26 ± 1.44c	32.79 ± 1.85a	25.02 ± 0.92a
	NaCl : CK	0.76	0.68	1.04	1.09

Values (mean ± standard errors) with the different letters in the same line are significantly different at *P* < 0.05 by Duncan's New Multiple Range Test. CK: 0 mmol·L^−1^ NaCl treatment; NaCl: 200 mmol·L^−1^ NaCl treatment.

**Table 4:** Effects of NaCl stress on Mg^2+^ content in different organs of *A. scoparia* and *A. vulgaris *“Variegate.”

Cultivars	Treatment	Mg^2+^ content (mg·g^−1^ dry weight)			
		Roots	Stems	Middle leaves	Upper leaves
*A. scoparia *	CK	2.63 ± 0.21c	2.10 ± 0.17b	5.63 ± 0.23a	4.77 ± 0.30a
	NaCl	0.74 ± 0.05d	2.20 ± 0.07b	6.06 ± 0.30a	5.60 ± 0.24a
	NaCl : CK	0.28	1.05	1.08	1.17

*A. vulgaris *“Variegate”	CK	14.88 ± 0.56a	4.92 ± 0.35a	4.55 ± 0.28a	4.23 ± 0.25a
	NaCl	9.31 ± 0.53b	5.31 ± 0.18a	4.93 ± 0.33a	4.98 ± 0.38a
	NaCl : CK	0.63	1.08	1.08	1.18

Values (mean ± standard errors) with the different letters in the same line are significantly different at *P* < 0.05 by Duncan's New Multiple Range Test. CK: 0 mmol·L^−1^ NaCl treatment; NaCl: 200 mmol·L^−1^ NaCl treatment.

**Table 5:** Effect of NaCl stress on the ratios of K^+^/Na^+^ in different parts of seedlings.

Materials	Treatment	Roots	Stems	Middle leaves	Upper leaves
*A. scoparia *	CK	6.85 ± 0.28a	4.56 ± 0.08b	4.81 ± 0.09a	8.81 ± 0.27b
	NaCl	0.38 ± 0.02c	0.82 ± 0.00c	0.86 ± 0.01c	0.90 ± 0.03d
*A. vulgaris *“Variegate”	CK	2.68 ± 0.18b	7.10 ± 0.54a	4.31 ± 0.23a	13.91 ± 0.24a
	NaCl	0.34 ± 0.03c	0.91 ± 0.03c	2.26 ± 0.17b	3.56 ± 0.09c

Values (mean ± standard errors) with the different letters in the same line are significantly different at *P* < 0.05 by Duncan's New Multiple Range Test. CK: 0 mmol·L^−1^ NaCl treatment; NaCl: 200 mmol·L^−1^ NaCl treatment.

**Table 6:** Effect of NaCl stress on absorbing and transporting selectivity ratios of K^+^ to Na^+^ in seedlings.

Materials	Treatment	Root absorption	From roots to stems	From stems to middle leaves	From stems to upper leaves
*A. scoparia *	CK	0.01	0.67	1.06	1.93
	NaCl	12.58	2.18	1.04	1.10
*A. vulgaris *“Variegate”	CK	0.00	2.64	0.61	1.98
	NaCl	11.38	2.65	2.48	3.93

CK: 0 mmol·L^−1^ NaCl treatment; NaCl: 200 mmol·L^−1^ NaCl treatment.

**Table 7:** Effect of NaCl stress on the ratios of Ca^2+^/Na^+^ in different parts of seedlings.

Materials	Treatment	Roots	Stems	Middle leaves	Upper leaves
*A. scoparia *	CK	2.30 ± 0.17b	1.82 ± 0.05b	2.43 ± 0.05a	2.74 ± 0.05b
	NaCl	0.17 ± 0.00d	0.32 ± 0.01c	0.52 ± 0.00c	0.38 ± 0.01d
*A. vulgaris *“Variegate”	CK	4.50 ± 0.31a	3.53 ± 0.41a	2.32 ± 0.15a	4.25 ± 0.36a
	NaCl	0.74 ± 0.05c	0.37 ± 0.04c	1.15 ± 0.07b	1.19 ± 0.05c

Values (mean ± standard errors) with the different letters in the same line are significantly different at *P* < 0.05 by Duncan's New Multiple Range Test. CK: 0 mmol·L^−1^ NaCl treatment; NaCl: 200 mmol·L^−1^ NaCl treatment.

**Table 8:** Effect of NaCl stress on absorbing and transporting selectivity ratios of Ca^2+^ to Na^+^ in seedlings.

Materials	Treatment	Root absorption	From roots to stems	From stems to middle leaves	From stems to upper leaves
*A. scoparia *	CK	0.01	0.80	1.34	1.50
	NaCl	8.68	1.85	1.61	1.17
*A. vulgaris *“Variegate”	CK	0.01	0.78	0.66	1.22
	NaCl	36.59	0.51	3.09	3.21

CK: 0 mmol·L^−1^ NaCl treatment; NaCl: 200 mmol·L^−1^ NaCl treatment.

**Table 9:** Effect of NaCl stress on the ratios of Mg^2+^/Na^+^ in different parts of seedlings.

Materials	Treatment	Roots	Stems	Middle leaves	Upper leaves
*A. scoparia *	CK	0.33 ± 0.03b	0.12 ± 0.01b	0.42 ± 0.00a	0.58 ± 0.03b
	NaCl	0.01 ± 0.00c	0.03 ± 0.00b	0.09 ± 0.01c	0.07 ± 0.03d
*A. vulgaris *“Variegate”	CK	1.07 ± 0.08a	0.55 ± 0.03a	0.33 ± 0.02ab	0.79 ± 0.06a
	NaCl	0.14 ± 0.00c	0.09 ± 0.00b	0.17 ± 0.02bc	0.24 ± 0.02c

Values (mean ± standard errors) with the different letters in the same line are significantly different at *P* < 0.05 by Duncan's New Multiple Range Test. CK: 0 mmol·L^−1^ NaCl treatment; NaCl: 200 mmol·L^−1^ NaCl treatment.

**Table 10:** Effect of NaCl stress on absorbing and transporting selectivity ratios of Mg^2+^ to Na^+^ in seedlings.

Materials	Treatment	Root absorption	From roots to stems	From stems to middle leaves	From stems to upper leaves
*A. scoparia *	CK	0.00	0.36	3.60	4.93
	NaCl	0.54	1.48	5.28	4.37
*A. vulgaris *“Variegate”	CK	0.00	0.51	0.61	1.44
	NaCl	6.91	0.65	1.86	2.56

CK: 0 mmol·L^−1^ NaCl treatment; NaCl: 200 mmol·L^−1^ NaCl treatment.
